# Supplementary material for: Combined Transplantation of Mesenchymal Progenitor and Neural Stem Cells to Repair Cervical Spinal Cord Injury
Source: Cells. 2025 Apr 23;14(9):630. doi: 10.3390/cells14090630 (PMC12072178; doi:10.3390/cells14090630)
Supplement: Supplementary file 1 [file cells-14-00630-s001.zip › cells-3551829-supplementary.pdf]

A Proportion of GFAP$^+$ DAPI

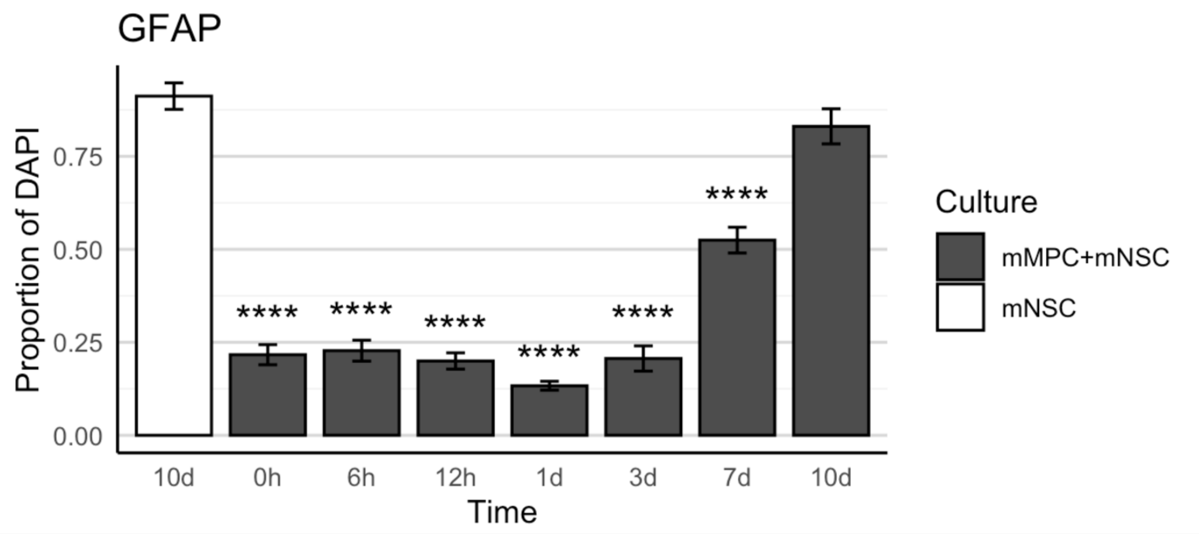

B Proportion of βIII-Tubulin$^+$ DAPI

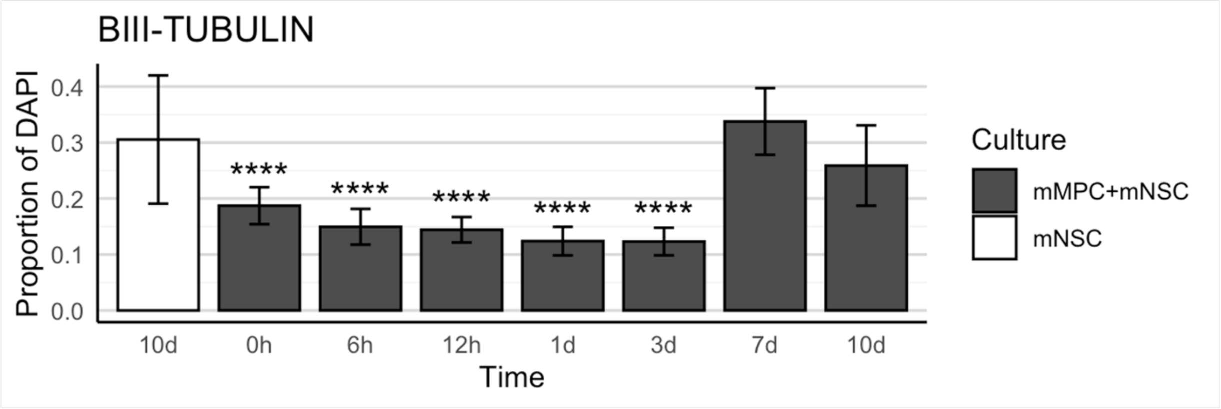

C **Proportion of MAP2⁺ DAPI**

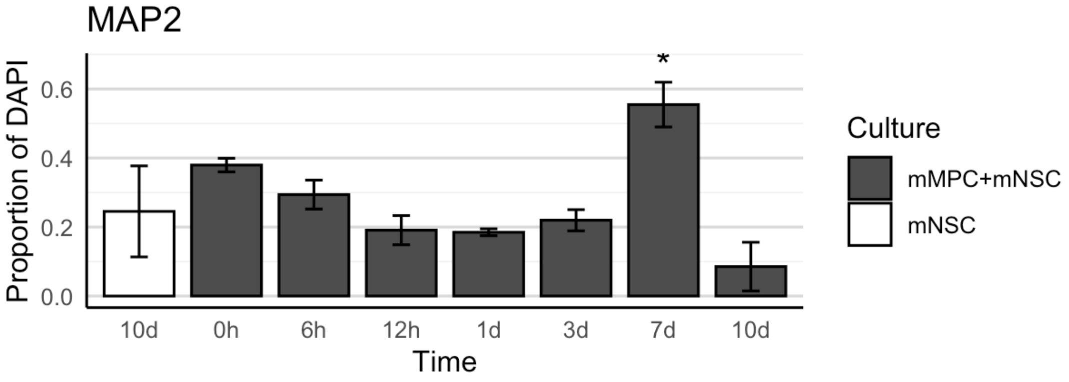

D **Proportion of O4⁺ DAPI**

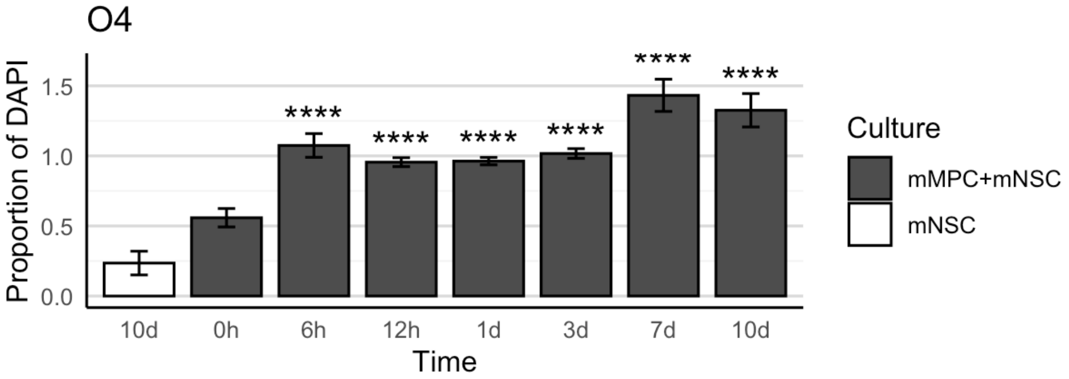

E Proportion of NG2+ DAPI

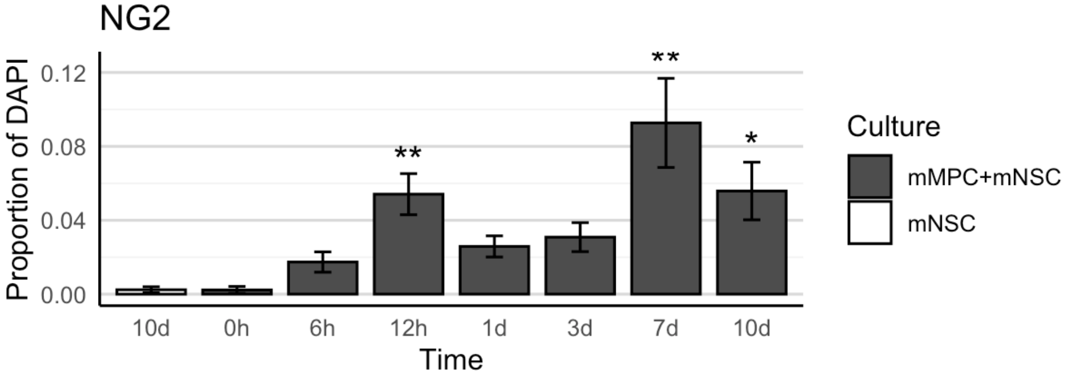

F Proportion of APC+ DAPI

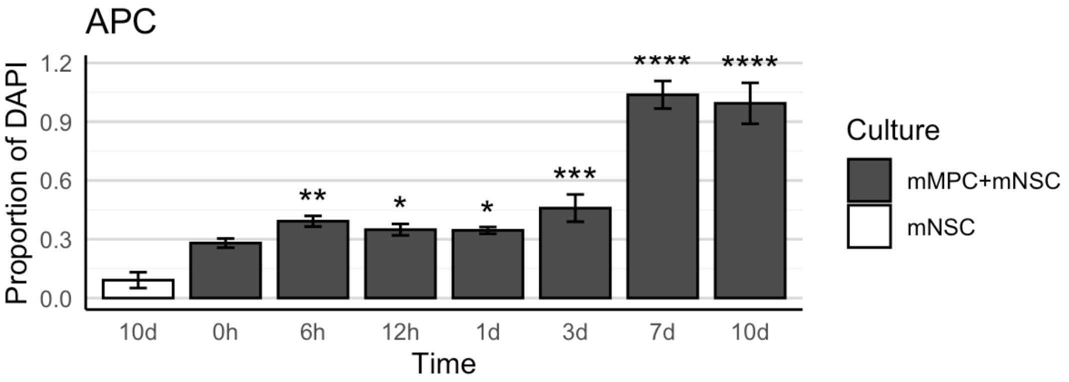

Supplemental Figure 1. ***In-Vitro*** **differentiation of NSC in MPC secretome**

Proportion of (A) GFAP+ (0h, p= 5.107x 10-15; 6h, p= 5.107x 10-15; 12h, p= 5.107x 10-15; 1d, p= 5.107x 10-15; 3d, p= 5.107x 10-15; 7d, p= 9.681 x 10-14), (B) βIII-Tubulin+ (0h, p= 1.571 x 10-5; 6h, p= 4.319 x 10-6; 12h, p= 3.994 x 10-6; 1d, p= 3.317 x 10-7; 3d, p= 5.540 x 10-7), (C) MAP2+ (7d, p= 1.295 x 10-2), (D) Tuj1+ (0h, p= 1.074 x 10-3; 12h, p= 2.023 x 10-2; 7d, p= 4.167 x 10-2), (E) O4+ (6h, p= 5.816 x 10-10; 12h, p= 3.555 x 10-8; 1d, p= 2.663 x 10-8; 3d, p= 3.029 x 10-9; 7d, p= 9.575 x 10-12; 10d, p= 1.379 x 10-11), (F) NG2+ (12h, p= 5.697 x 10-3; 1d, p= 2.377 x 10-1; 7d, p= 1.800 x 10-3; 10d, p= 1.965 x 10-2), and (G) APC+ (6h, p= 2.595 x 10-3; 12h, p= 3.132 x 10-2; 1d, p= 1.087 x 10-2; 3d, p= 3.945 x 10-4; 7d, p= 0.0000; 10d, p= 0.0000) DAPI+ Nuclei. All groups listed are in mMPC + mNSC cultures, and the statistics are computed in comparison to the 10-day mNSC-only group.
